# Viral Kinetics of Severe Acute Respiratory Syndrome Coronavirus 2 in Patients with Coronavirus Disease 2019

**DOI:** 10.1128/Spectrum.00793-21

**Published:** 2021-10-27

**Authors:** Da Young Kim, Eun Kyung Bae, Jun-Won Seo, Na Ra Yun, Choon-Mee Kim, Dong-Min Kim

**Affiliations:** a Department of Internal Medicine, College of Medicine, Chosun University, Gwangju, Republic of Korea; b Department of Premedical Science, College of Medicine, Chosun University, Gwangju, Republic of Korea; Keck School of Medicine of the University of Southern California

**Keywords:** COVID-19, SARS-CoV-2, viral kinetics

## Abstract

To determine the relationship between viral kinetics and severity of disease in severe acute respiratory syndrome coronavirus 2 (SARS-CoV-2) infection, we investigated the viral kinetics and compared the viral loads of patients with coronavirus disease 2019 (COVID-19; the disease caused by SARS-CoV-2), stratified by symptoms and severity. We determined the viral kinetics of 100 patients diagnosed with COVID-19 at Chosun University Hospital between February 2020 and May 2021 and analyzed the differences between asymptomatic, symptomatic, and nonsurvivor patients and between patients who died and those who survived. Clinical samples, comprising respiratory specimens (sputum samples and nasopharynx and oropharynx swab samples), were obtained at different time points of hospitalization, at 1, 3 to 5, 7, 10, 14, and 30 days. SARS-CoV-2 was detected using real-time reverse transcription-PCR (RT-PCR). All three groups, asymptomatic, symptomatic, and deceased patients, had higher numbers of viral copies at symptom onset, and the asymptomatic group had lower numbers of viral copies than the symptomatic or nonsurvivor group. Viral RNA release was detected until 30 days after symptom onset. The virus cleared up earlier in asymptomatic patients than in symptomatic and nonsurvivor patients, and it cleared up earlier in mildly affected patients than in severely affected patients. The cycle threshold values tended to be significantly lower in the group receiving steroids than in the nonsteroid group, even in the low-risk group with a pneumonia severity index of less than 90. The viral loads in patients with COVID-19 were significantly different according to disease severity and steroid use.

**IMPORTANCE** In our study, we analyzed the viral kinetics of COVID-19 patients. Our study reveals differences in viral shedding according to the severity of disease in COVID-19 patients. Viral shedding had a longer duration in severely affected patients, and the cyclic threshold values were lower in the group receiving steroids. This study is expected to be helpful in analyzing the trend of the disease course according to steroid use and severity of SARS-CoV-2 disease.

## INTRODUCTION

The World Health Organization declared a pandemic in March 2020 after the initial emergence of an infectious coronavirus disease (coronavirus disease 2019 [COVID-19]) caused by severe acute respiratory syndrome coronavirus 2 (SARS-CoV-2) in Wuhan, Hubei Province, China, in December 2019 (1). In South Korea, as of 14 September 2021, there has been a cumulative total of 275,910 confirmed COVID-19 cases and 2,367 deaths (2). An understanding of viral kinetics is essential to prevent the spread of COVID-19 and to provide adequate treatment.

Therefore, to determine the relationship between viral kinetics and severity of SARS-CoV-2 infection, we determined the viral kinetics and compared the viral loads among COVID-19 patients based on symptoms and severity.

## RESULTS

The median age of patients in the nonsurvivor group was 84 years (interquartile range [IQR], 79.6 to 90), whereas that of the survivor group was lower, at 64.50 years (IQR, 51 to 75.8). Of the 100 patients, 48 (48%) were men and 52 (52%) were women. Hypertension, diabetes, and cardiovascular diseases were the most common underlying diseases. Fever was the most common clinical symptom in both the survivor and nonsurvivor cases, followed by coughing and dyspnea.

Approximately 83% of patients in the nonsurvivor group were administered oxygen at the time of admission, and 100% of these patients received oxygen during the hospitalization period (*P = *0.001). Conversely, in the survivor group, 34% of patients received oxygen at the time of admission, and 45.5% received oxygen during hospitalization (*P = *0.001). During hospitalization, mechanical ventilation was applied to 67% of patients in the nonsurvivor group but to only 7% of patients in the survivor group (*P < *0.001). Antiviral agents were administered to 92% of patients in the nonsurvivor group and to only 33% of patients in the survivor group (*P = *0.001). The severely affected group had more underlying diseases, such as hypertension, diabetes, and cardiovascular disease, than the mildly affected group (*P* = 0.003, *P* = 0.001, and *P* = 0.046, respectively).

In the severely affected group, 65.4% had fever during hospitalization, whereas only 22.9% of the mildly affected group had fever (*P < *0.001). Antiviral agents were administered in 84.6% of the severely affected group but in only 6.3% of the mildly affected group (*P < *0.001) (Table 1).

**TABLE 1:** Patient information of individuals in the mildly and severely affected groups

Characteristic	Value for^*a*^:			
	Total (*n* = 100)	Mildly affected (*n* = 48)	Severely affected (*n* = 52)	*P* value
Age (yr)	66 (53–79.8)	53.5 (28–36.8)	78.5 (65–84)	0.678
Sex (male/female)	48/52 (48/52)	22/26 (46/54)	26/26 (50/50)	<0.001
Diabetes	25 (25)	5 (10.4)	20 (38.5)	0.001
Hypertension	40 (40)	12 (25)	28 (53.8)	0.003
Cardiovascular disease	16 (16)	4 (8.3)	12 (23.1)	0.046
Chronic lung disease	2 (2)	0	2 (3.8)	0.172
Chronic kidney disease	2 (2)	0	2 (3.8)	0.172
Cancer	8 (8)	4 (8.3)	4 (7.7)	0.906
Former or current smoker	5 (5)	4 (8.3)	1 (1.9)	0.977
Fever	45 (45)	11 (22.9)	34 (65.4)	<0.001
Cough	33 (33)	17 (35.4)	16 (30.8)	0.623
Dyspnea	17 (17)	0	17 (32.7)	<0.001
Sore throat	12 (12)	8 (16.7)	4 (7.7)	0.170
Headache	9 (9)	5 (10.4)	4 (7.7)	0.636
Myalgia	16 (16)	7 (14.6)	9 (17.3)	0.712
Nausea or vomiting	6 (6)	1 (2.1)	5 (9.6)	0.115
Diarrhea	7 (7)	4 (8.3)	3 (5.8)	0.617
Mechanical ventilation	14 (14)	0	14 (26.9)	<0.001
Antiviral agent	47 (47)	3 (6.3)	44 (84.6)	<0.001
Lopinavir/ritonavir (Kaletra)	7 (7)	2 (4.2)	5 (9.6)	0.288
Hydroxychloroquine (Oxiclorin)	3 (3)	1 (2.1)	2 (3.8)	0.607
Remdesivir (Veklury)	40 (40)	0	40 (76.9)	<0.001

a Age, sex, underlying disease, and clinical symptoms are expressed as the median value ± interquartile range (IQR) or percentage, and the number of viral copies and chest score are expressed as the mean value (standard error of the mean [SEM]).

The median ages of asymptomatic, symptomatic, and nonsurvivor patients were 37 years (IQR, 32 to 49), 66 years (IQR, 56 to 78), and 84 years (IQR, 79.5 to 90) (*P < *0.001). None of the asymptomatic patients had underlying hypertension, whereas the incidence was 41.8% in the symptomatic group and 58.3% in the nonsurvivor group (*P = *0.021). All nonsurvivors required oxygen therapy, while only 50.6% of symptomatic patients received oxygen therapy (*P < *0.001) (Table 2).

**TABLE 2:** Clinical information of asymptomatic, symptomatic, and nonsurvivor patients

Characteristic	Value for^*a*^:				
	Total (*n* = 100)	Asymptomatic (*n* = 9)	Symptomatic (*n* = 79)	Nonsurvivor (*n* = 12)	*P* value
Age (yr)^*b*^	66 (53–79.8)	37 (32–49)	66 (56–78)	84 (79.5–90)	<0.001
Sex (male/female)	48/52 (48/52)	5/4 (56/44)	38/41 (48/52)	5/7 (42/58)	0.821
Diabetes	25 (25)	0	20 (25.3)	5 (41.7)	0.094
Hypertension	40 (40)	0	33 (41.8)	7 (58.3)	0.021
Cardiovascular disease	16 (16)	0	14 (17.7)	2 (16.7)	0.392
Chronic lung disease	2 (2)	0	1 (1.3)	1 (8.3)	0.243
Chronic kidney disease	2 (2)	0	2 (2.5)	0	0.764
Cancer	8 (8)	0	7 (8.9)	1 (8.3)	0.652
Former or current smoker	5 (5)	3 (33.3)	2 (2.5)	0	0.015
Fever	45 (45)	0	37 (46.8)	8 (66.7)	0.008
Cough	33 (33)	0	29 (36.7)	4 (33.3)	0.087
Dyspnea	17 (17)	0	13 (16.5)	4 (33.3)	0.130
Sore throat	12 (12)	0	12 (15.2)	0	0.166
Headache	9 (9)	0	9 (11.4)	0	0.272
Myalgia	16 (16)	0	16 (20.3)	0	0.082
Nausea or vomiting	6 (6)	0	5 (6.3)	1 (8.3)	0.705
Diarrhea	7 (7)	0	6 (7.6)	1 (8.3)	0.689
Oxygen requirement	52 (52)	0	40 (50.6)	12 (100)	<0.001
Mechanical ventilation	14 (14)	0	6 (7.6)	8 (66.7)	<0.001
Antiviral agent	47 (47)	0	36 (45.6)	11 (91.7)	<0.001
Lopinavir/ritonavir (Kaletra)	7 (7)	0	7 (8.9)	0	0.371
Hydroxychloroquine (Oxiclorin)	3 (3)	0	3 (3.8)	0	0.666
Remdesivir (Veklury)	40 (40)	0	29 (36.7)	11 (91.7)	<0.001

a Age, sex, underlying disease, and clinical symptoms are expressed as the median value ± interquartile range (IQR) or percentage, and the number of viral copies and chest score are expressed as the mean value (standard error of the mean [SEM]).

b The mean age of patients in the asymptomatic, symptomatic, and nonsurvivor groups was different between the groups. Of note, the mean difference between the groups was 41.5 years, which was the largest difference between the asymptomatic and nonsurvivor groups.

The median pneumonia severity index (PSI) scores were 94 (IQR, 78 to 112) in the nonsurvivor group and 62 (IQR, 44 to 80) in the survivor group (*P* < 0.001). At the time of admission, the cycle threshold (*C_T_*) value for samples from the upper respiratory tract was found to be significantly lower in the nonsurvivor group than in the survivor group (*P = *0.009 and *P = *0.008).

**FIG 1:**
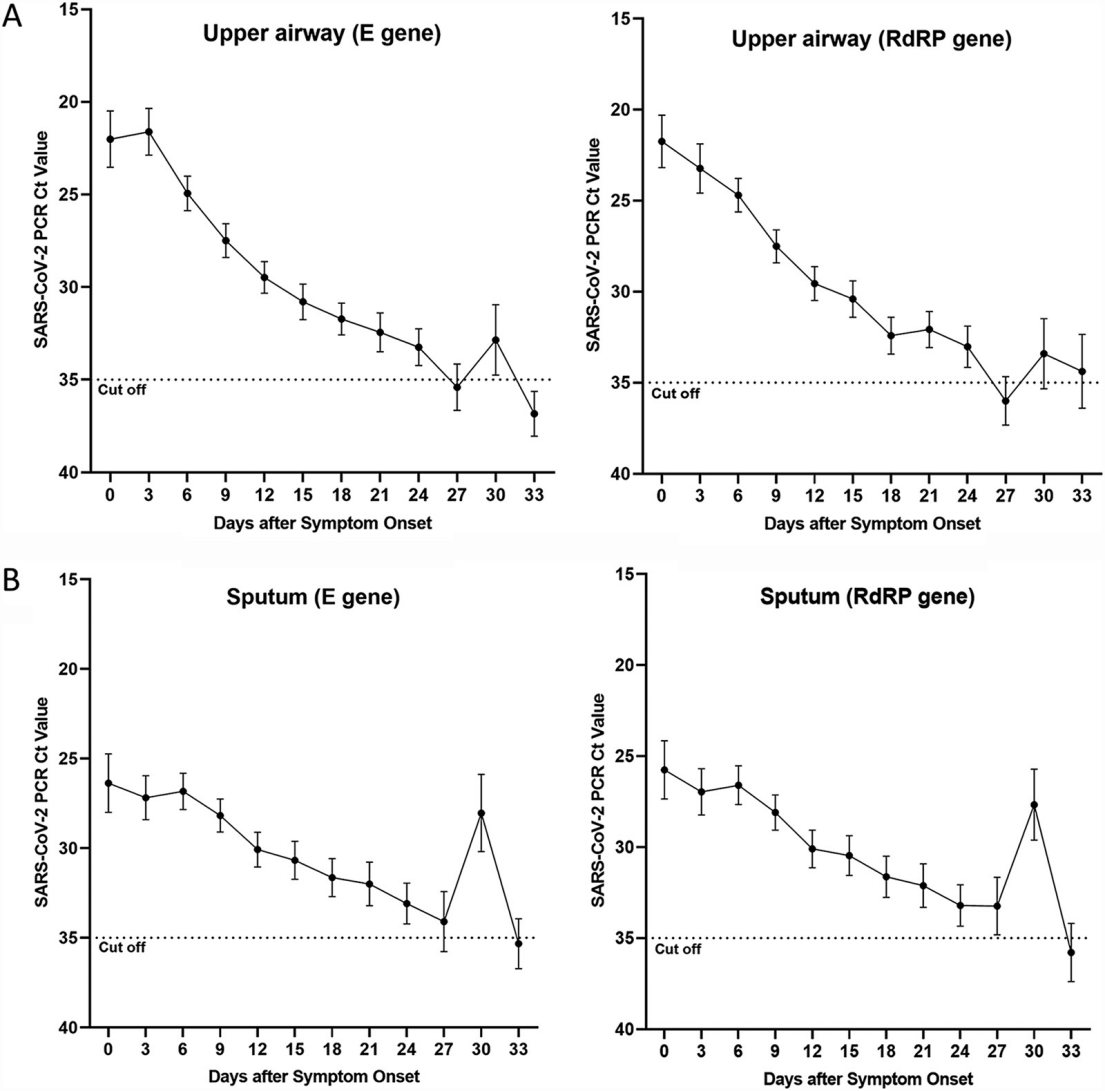
(A) Cycle threshold values of *RdRP* and *E* targeted in nasopharyngeal swab samples to detect severe acute respiratory syndrome coronavirus 2 (SARS-CoV-2) by the number of days after symptom onset in all patients. (B) Cycle threshold values when *RdRP* and *E* were targeted in sputum specimens to detect severe acute respiratory syndrome coronavirus 2 (SARS-CoV-2) on the basis of the days after symptom onset in all patients. Samples taken on days −2 to 1 were included in day 0 of the symptom occurrence criteria. In addition, the 3rd day included days 2 to 4, the 6th day included days 5 to 7, the 9th day included days 8 to 10, the 12th day included days 11 to 13, the 15th day included days 14 to 16, and the 18th day included days 17 to 19. It was decided to include specimens from the 20th to 22nd day for the 21st day, from the 23rd to 25th day for the 24th day, from the 26th to 28th day for the 27th day, from the 29th to 31st day for the 30th day, and from the 32nd to 34th day for the 33rd day. In the case of asymptomatic patients, the date of confirmation of COVID-19 was used as the date of symptom onset.

**FIG 2:**
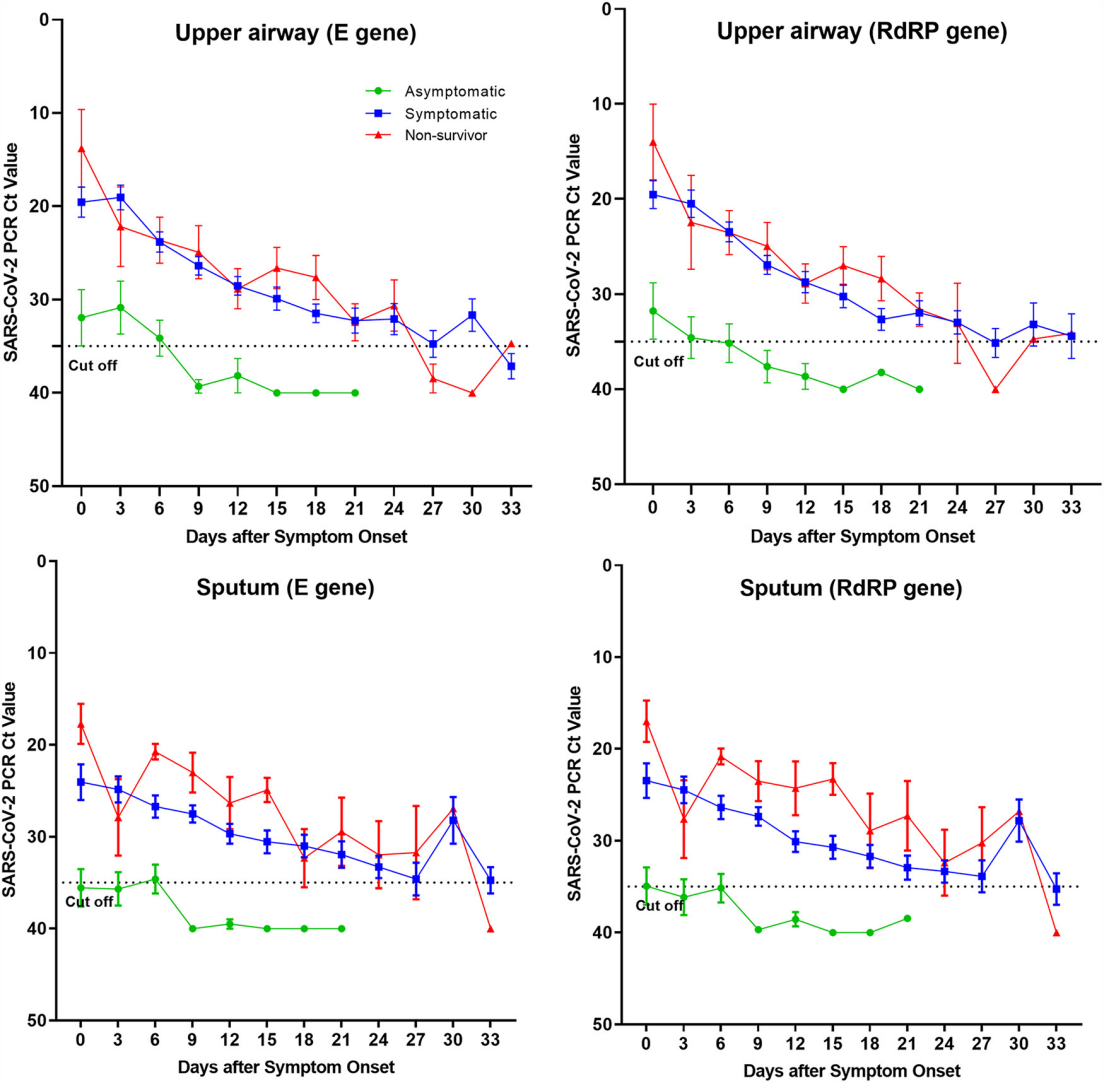
Cycle threshold values of severe acute respiratory syndrome coronavirus 2 (SARS-CoV-2) in asymptomatic, symptomatic, and nonsurvivor patients, according to the onset of symptoms. Days of sample collection are described in the legend to Fig. 1.

When the cutoff *C_T_* value was set to 35 for all hospitalized patients, viral RNA shedding was detected until 30 days after symptom onset (Fig. 1A and B). All three groups, i.e., asymptomatic, symptomatic, and nonsurvivor groups, tended to have a higher viral copy number at the beginning of symptom onset, and the asymptomatic group showed a tendency to have a lower viral copy number than the symptomatic or nonsurvivor group (Fig. 2).

**FIG 3:**
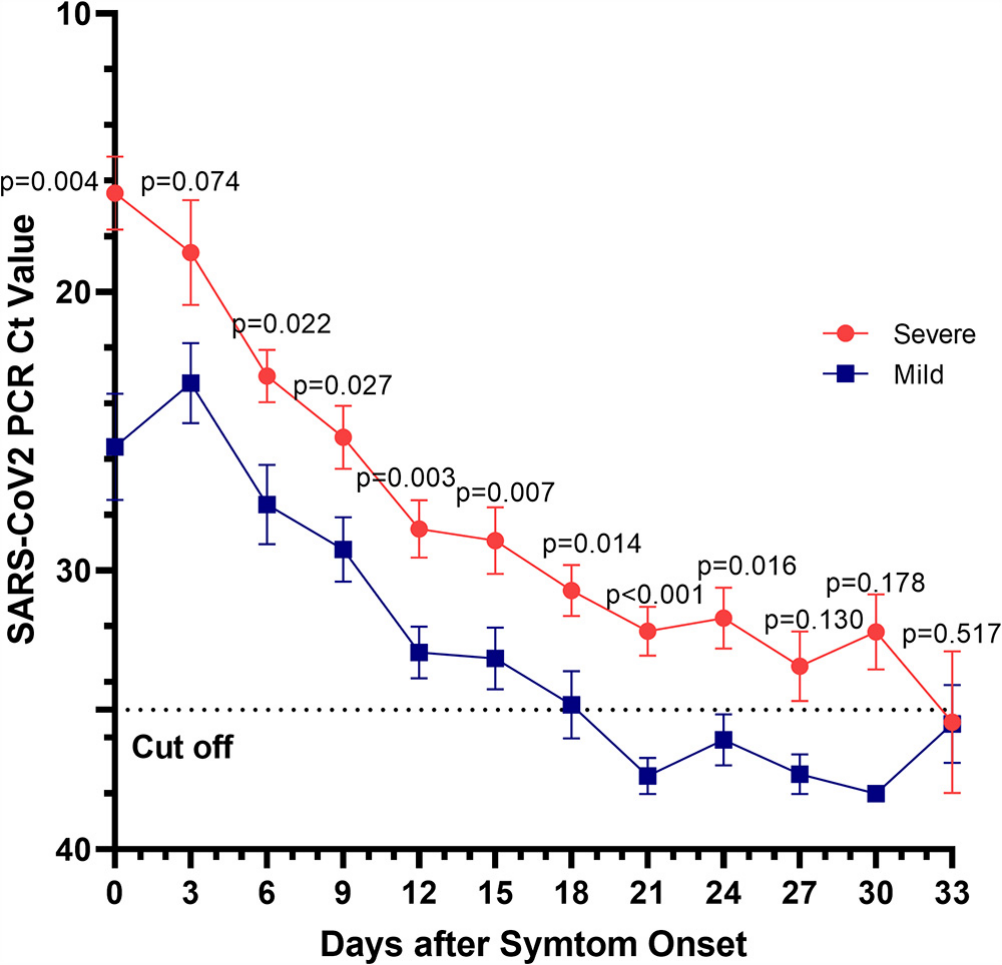
Cycle threshold values when the *E* gene was targeted using nasopharyngeal swab samples to detect severe acute respiratory syndrome coronavirus 2 (SARS-CoV-2) on the basis of the days after symptom onset in severely affected (group administered oxygen) and mildly affected (group not administered oxygen) patients. Days of sample collection are described in the legend to Fig. 1.

In both the severely and mildly affected groups, the number of viral copies was highest before or at the beginning of symptom onset, and the viral load tended to decrease over time. Except for the 3rd day from symptom onset, there were statistically significant differences in the *C_T_* values between the two groups within 24 days of symptom onset. The severely affected group showed a peak in the number of viral copies at the time of admission, and the mildly affected group showed a peak on the 3rd day of symptom onset (Fig. 3).

**FIG 4:**
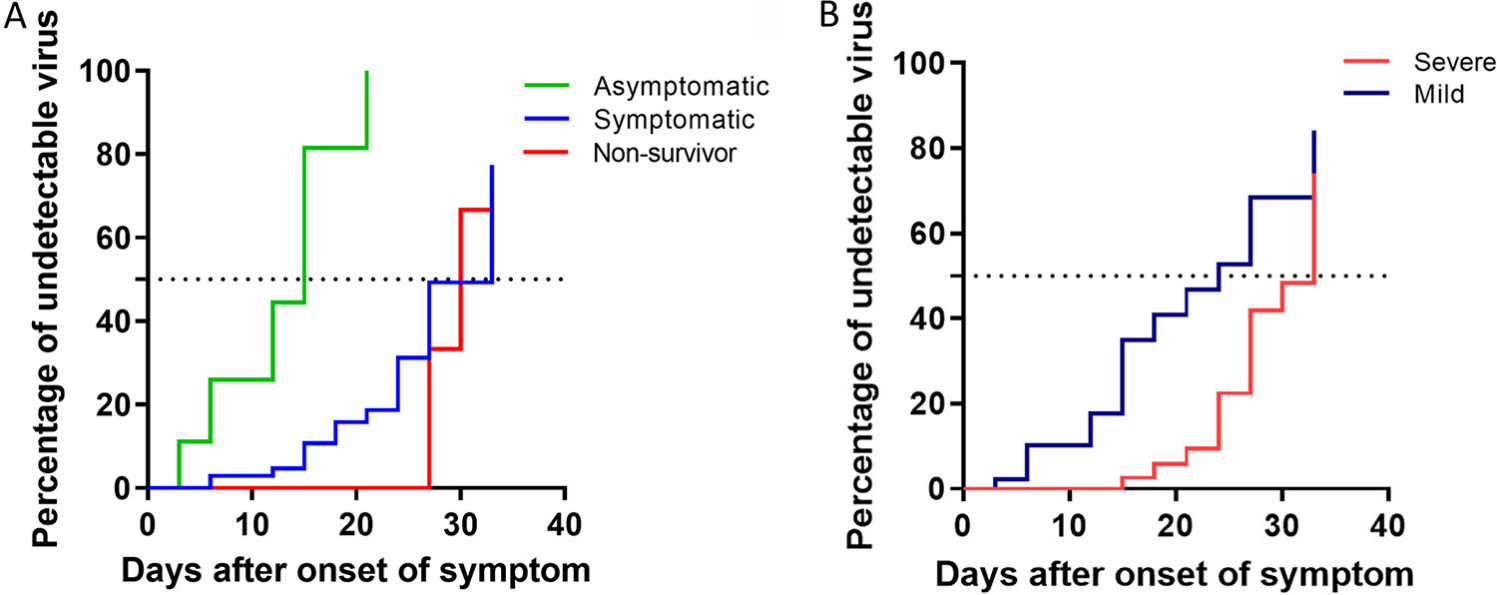
(A) Kaplan-Meier curve showing the time to viral RNA clearance for asymptomatic, symptomatic, and nonsurvivor coronavirus disease 2019 (COVID-19) patients when the *E* gene was targeted in nasopharyngeal swab samples. (B) Kaplan-Meier curve indicates time to viral RNA clearance for patients with mild and severe coronavirus disease 2019 (COVID-19) when the *E* gene was targeted by using nasopharyngeal swab samples. Days of sample collection are described in the legend to Fig. 1.

The average viral shedding durations of SARS-CoV-2 virus were 15, 33, and 30 days in the asymptomatic, symptomatic, and nonsurvivor groups, respectively (Fig. 4A). In addition, the average viral shedding was 33 and 24 days in the severely and mildly affected groups, respectively (Fig. 4B). In the case of the nonsurvivor group, there were very few cases in which the virus was undetectable before about 4 weeks from symptom onset. In the mildly affected group, the percentage of those with an undetectable viral load gradually increased with time from the onset of symptoms. In the severely affected group, there were few cases in which the virus was undetectable until almost 2 weeks from symptom onset (Fig. 4A and B).

**FIG 5:**
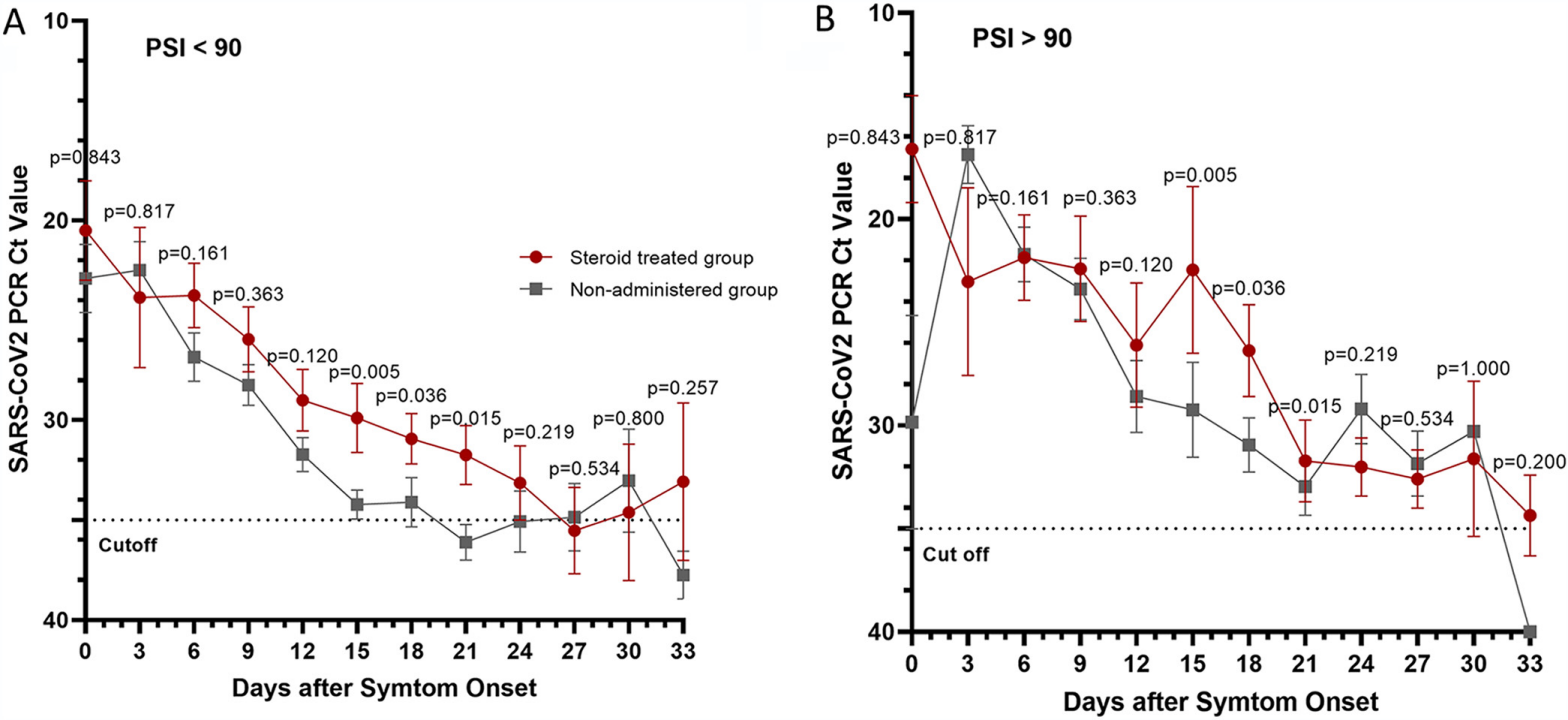
(A) Cycle threshold values when the *E* gene was targeted in nasopharyngeal swab samples to detect severe acute respiratory syndrome coronavirus 2 (SARS-CoV-2) according to the number of days after symptom onset in steroid-treated group and non-steroid-treated (non-administered) group in patients with pneumonia severity index (PSI) below 90. (B) Cycle threshold values when the *E* gene was targeted using nasopharyngeal swab samples to detect severe acute respiratory syndrome coronavirus 2 (SARS-CoV-2) on the basis of the days after symptom onset in steroid-treated group and non-steroid-treated (non-administered) group in patients with pneumonia severity index (PSI) of 90 or higher. Days of sample collection are described in the legend to Fig. 1.

In the group with a PSI of less than 90, the *C_T_* value tended to be significantly lower in the group receiving steroids on days 15, 18, and 21 after symptom onset than in the nonsteroid group (Fig. 5A). In the group with a PSI of 90 or higher, the *C_T_* value tended to be significantly lower in the steroid-treated group on days 15, 18, and 21 after symptom onset than in the nonsteroid group (Fig. 5B).

**FIG 6:**
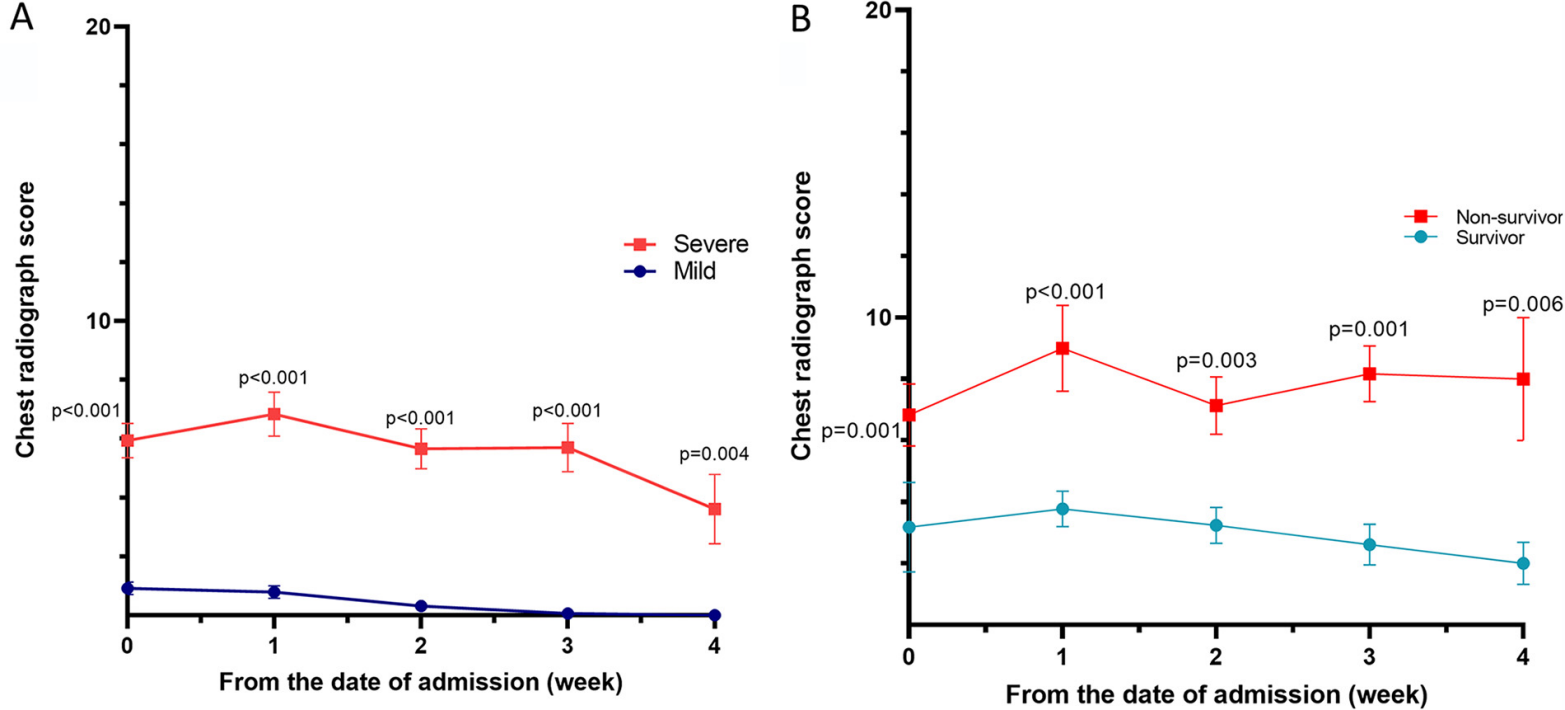
(A) Chest radiograph scores in severely and mildly affected groups. (B) Chest radiograph scores in the nonsurvivor and survivor groups.

The chest radiography score was calculated and analyzed by dividing the cohort into the 48 patients who did not receive oxygen therapy (room air) and the 52 patients who received oxygen therapy (low-flow nasal cannula, high-flow nasal cannula, or mechanical ventilator). The radiographs were analyzed on the day of admission and at weeks 1, 2, 3, and 4 of hospitalization; we found that the chest score tended to be lower in the mildly affected group than in the severely affected group (Fig. 6A). Similarly, the chest score tended to be lower in the survivor group than in the nonsurvivor group (Fig. 6B).

## DISCUSSION

Wang et al. reported that SARS-CoV-2 RNA was maintained for 42 days in pharyngeal and sputum samples (3). In the case of patients in our hospital, viral shedding was maintained until the 18th day from symptom onset in the mildly affected group, and shedding continued until the 33rd day from symptom onset in the severely affected group. The severely affected group showed a peak in the number of viral copies at the time of admission, and the mildly affected group showed a peak on the 3rd day from symptom onset. In a study by Yilmaz et al., the viral load was high in both the mildly and severely affected groups at the initial stage of symptom onset, as in our study, but, unlike in our study, the duration of viral shedding did not differ according to severity (4).

In the study by Jang et al., the viral load was high at symptom onset and decreased after symptom onset. Specifically, rapid viral proliferation was observed between 0 and 6 days before symptom onset, and the viral load was higher before symptom onset and at the early stage (5). In the study by To et al., the SARS-CoV-2 viral load peaked at the beginning of the onset of symptoms, unlike other respiratory viruses (6). In the study by He et al., the viral load peaked before and after the onset of symptoms (7). In our study, the *C_T_* value was maintained below 30 from the onset of symptoms to the 12th day, whereas in the study by Jang et al., the *C_T_* value was maintained below 30 from the onset of symptoms to the 10th day (5).

In the study by Kwon et al., the durations of positive RT-PCR results were significantly shorter in the asymptomatic and mildly affected groups than in the severely affected and critical groups. The median time (IQR) from the onset of symptoms to negative conversion of RT-PCR results for SARS-CoV-2 was on day 18 (14 to 24) (8).

Considering the information in the literature regarding a number of reported cases of severe acute respiratory syndrome (SARS), the viral load increased from the early phase of the disease after symptom onset and peaked after approximately 10 days. The viral load then gradually decreased, and virus shedding was observed from day 10 to day 21 (9). In another study on SARS, the viral load in nasopharyngeal swabs peaked on day 10 after symptom onset. The serum viral loads showed a proportional relationship with oxygen saturation reduction, mechanical breathing, and death (10).

Studies involving patients with Middle East respiratory syndrome (MERS) have also shown that the mortality rate in patients with MERS increased with a high viral load (11). In addition, in studies on the viral load and severity of MERS-CoV, a lower *C_T_* value in the sample taken from the upper respiratory tract was associated with a higher mortality rate and higher ICU hospitalization rate (12). Another study showed that the peak and mean viral loads tended to be similar between patients with MERS and those with SARS (13).

A high viral load in the upper respiratory tract is associated with disease severity in other respiratory viruses (e.g., respiratory syncytial virus [RSV]) (14). In a study by Lee et al., plasma SARS-CoV RNA concentrations were higher in steroid-treated patients than in placebo-treated patients in the 2nd and 3rd weeks from symptom onset. The median times at which SARS-CoV was not detected in plasma were also different, at 12 days (11 to 20 days) in the steroid-treated group and 8 days (8 to 15 days) in the placebo-treated group (15). In our study, the *C_T_* values were significantly lower in the steroid-treated group than in the non-steroid-treated group on days 15, 18, and 21 from symptom onset.

In a study on patients with SARS, 60 of 75 patients (80%) showed radiological worsening after a mean of 7.4 days (9). In another study of 17 patients with MERS, chest radiograph scores peaked at approximately 2 weeks after symptom onset (16).

In our study, the chest score tended to be the highest in the severely affected group 1 week after the onset of symptoms; the mildly affected group had the highest score on the day of admission. One week after symptom onset, both the nonsurvivor and the survivor group had the highest chest scores and pneumatic infiltration tended to be the most severe.

This study has a few limitations; first, the sample size was small at only 100 patients, and it was a single-hospital study.

### Conclusion.

In this study, we showed the variations from peak to trough of the kinetics of the novel coronavirus. The viral load in COVID-19 patients was high at the onset of symptoms. Viral shedding differed according to severity and steroid use. In particular, the duration of viral shedding was longer in the symptomatic, severe, and nonsurvivor groups than in mild survivors. Based on the results of our study, understanding the viral kinetics is important to identify critical patients and formulate treatment strategies. This study is expected to be helpful in analyzing the trends of the disease course according to the symptoms and severity of SARS-CoV-2 infection.

## MATERIALS AND METHODS

### Study approval.

This study was approved by the Institutional Review Board (IRB) of Chosun University Hospital, and informed consent was obtained from all patients or their legal guardians. This retrospective study utilized the cohort study of COVID-19 patients (IRB no. 2020-04-003) with premade case record forms from February 2020 to May 2021. Written consent to participate was obtained from all participants, in accordance with the tenets of the Declaration of Helsinki. All methods were performed in accordance with relevant South Korean guidelines and regulations.

### Patients and study site.

Chosun University Hospital, a 910-bed hospital, is located in Gwangju, South Korea. We determined the viral kinetics and chest radiographs of 100 patients diagnosed with COVID-19 at Chosun University Hospital between February 2020 and May 2021. We analyzed the differences in viral kinetics between 9 asymptomatic, 74 symptomatic, and 12 nonsurvivor patients, as well as between the 12 nonsurvivor and 88 surviving patients. These patients were then divided into two groups based on the demand for oxygen therapy. Forty-eight patients who did not receive oxygen therapy, including asymptomatic patients, were classified into the mildly affected group and were compared with the 52 patients who received oxygen therapy, classified as the severely affected group. In order to reduce selection bias due to the high probability of steroid administration in severely affected patients, viral kinetics were analyzed according to the severity of steroid administration by dividing those who received steroids into two groups using the pneumonia severity index (PSI). A PSI of 90 or higher was considered the high-risk group, and a PSI of less than 90 was considered the low-risk group.

### Diagnostic methods.

To extract the viral nucleic acid, we collected the nasopharyngeal and oropharyngeal swab samples using commercial UTM kits containing 1 ml of viral transport medium (Noble Bio, Seoul, South Korea). Sputum samples from the patients were diluted in phosphate-buffered saline (PBS), mixed, and centrifuged (200 × *g* for 1 min), and the supernatant was subjected to RNA extraction. The viral RNA was extracted using a fully automated instrument (BioSewoom, South Korea) with the real-prep viral DNA/RNA kit (Biosewoom, South Korea).

Clinical samples, comprising respiratory specimens (sputum samples and nasopharynx and oropharynx swab samples), were analyzed at different time points of hospitalization, at 1, 3 to 5, 7, 10, 14, and 30 days.

SARS-CoV-2 was detected using real-time reverse transcription-PCR (RT-PCR). RNA was extracted from nasopharyngeal swab samples to detect SARS-CoV-2. RNA in the sputum specimens was quantified using the RT-PCR assay. The main target genes for the identification of SARS-CoV-2 are open reading frame 1a (ORF 1a) and ORF 1b, the RNA-dependent RNA polymerase (RdRp) gene, the envelope (E) gene, and the nucleocapsid (N) gene (17).

Two products approved in South Korea that detect two of the target genes (the E and RdRp genes), the Powerchek 2019-nCoV real-time PCR kit (Kogenebiotech, Seoul, South Korea) and the Standard M n-CoV real-time detection kit (SD Biosensor, Suwon, South Korea), were used for testing with a cutoff cycle threshold (*C_T_*) value of >35 cycles. We analyzed chest radiographs on the day of hospitalization and later at weeks 1, 2, 3, and 4 of hospitalization to monitor and quantify the severity of lung involvement. Chest radiograph scores were calculated using 323 chest radiographs obtained from 100 patients and were obtained by dividing each lung into the upper, middle, and lower zones and scoring each zone from 0 to 4 points based on the degree of infiltration. The scores for each lung were then added considering a total of six zones per patient, yielding a total score ranging from 0 to 24 (18).

### Statistical analysis.

All data were analyzed using SPSS (version 18.0; SPSS, Inc., Chicago, IL, USA). Age, sex, underlying disease, and clinical symptoms are expressed as the median value ± interquartile range (IQR) or percentage, and the number of viral copies and chest score are expressed as the mean value (standard error of the mean [SEM]). The Mann-Whitney U test was used to determine the average number of viral copies and chest score for each time point. To analyze the differences in clinical information between asymptomatic, symptomatic, and nonsurvivor groups, the Kruskal-Wallis test was performed. When comparing viral RNA clearance according to severity and symptom status using the Kaplan-Meier curve, a log-rank test was performed. Information on sex, underlying disease, symptoms, and presence or absence of oxygen therapy among asymptomatic, symptomatic, and nonsurvivor groups was analyzed using the chi-square test. The mean ages between the asymptomatic, symptomatic, and nonsurvivor groups were tested using analysis of variance (ANOVA) and Scheffe *post hoc* tests. Statistical significance was set at a *P* value of <0.05.
